# Hyperpolarized ketone body metabolism in the rat heart

**DOI:** 10.1002/nbm.3912

**Published:** 2018-04-10

**Authors:** Jack J. Miller, Daniel R. Ball, Angus Z. Lau, Damian J. Tyler

**Affiliations:** ^1^ Department of Physiology, Anatomy and Genetics University of Oxford Oxford UK; ^2^ Department of Physics University of Oxford Oxford UK; ^3^ Sunnybrook Research Institute Imaging Research Toronto ON Canada; ^4^ Department of Medical Biophysics University of Toronto Toronto ON Canada

**Keywords:** ^13^C, acetoacetate, hyperpolarization, ketone bodies

## Abstract

The aim of this work was to investigate the use of ^13^C‐labelled acetoacetate and β‐hydroxybutyrate as novel hyperpolarized substrates in the study of cardiac metabolism. [1‐^13^C]Acetoacetate was synthesized by catalysed hydrolysis, and both it and [1‐^13^C]β‐hydroxybutyrate were hyperpolarized by dissolution dynamic nuclear polarization (DNP). Their metabolism was studied in isolated, perfused rat hearts. Hyperpolarized [1‐^13^C]acetoacetate metabolism was also studied in the *in vivo* rat heart in the fed and fasted states. Hyperpolarization of [1‐^13^C]acetoacetate and [1‐^13^C]β‐hydroxybutyrate provided liquid state polarizations of 8 ± 2% and 3 ± 1%, respectively. The hyperpolarized T_1_ values for the two substrates were 28 ± 3 s (acetoacetate) and 20 ± 1 s (β‐hydroxybutyrate). Multiple downstream metabolites were observed within the perfused heart, including acetylcarnitine, citrate and glutamate. In the *in vivo* heart, an increase in acetylcarnitine production from acetoacetate was observed in the fed state, as well as a potential reduction in glutamate. In this work, methods for the generation of hyperpolarized [1‐^13^C]acetoacetate and [1‐^13^C]β‐hydroxybutyrate were investigated, and their metabolism was assessed in both isolated, perfused rat hearts and in the *in vivo* rat heart. These preliminary investigations show that DNP can be used as an effective *in vivo* probe of ketone body metabolism in the heart.

**Abbreviations used**
DMSO: dimethyl sulphoxide
DNP: dynamic nuclear polarization
DOTA: 1,4,7,10‐tetraazacyclododecane‐1,4,7,10‐tetraacetic acid
ECG: electrocardiogram
Gd: gadolinium
HMBC: heteronuclear multiple bond correlation
HR: heart rate
KH: Krebs–Henseleit
LVDP: left ventricular developed pressure
MRI: magnetic resonance imaging
NMR: nuclear magnetic resonance
RF: radiofrequency
RPP: rate–pressure product
SNR: signal‐to‐noise ratio
TCA: tricarboxylic acid

## 1 INTRODUCTION

The ketone bodies acetoacetate and 3‐β‐hydroxybutyrate act as important energy sources during starvation.^1^ Ketone bodies are produced from fatty acid metabolism in the liver under low‐carbohydrate conditions, and their subsequent metabolism plays a key role in sparing glucose utilization and reducing proteolysis.^2^, ^3^ This is important, for example, in the maintenance of brain function during low blood glucose conditions, and the use of ketone bodies accounts for nearly two‐thirds of the brain's energy usage during prolonged fasting and starvation.^4^ As a fuel source, ketone bodies have thermodynamically favourable characteristics, such as oxygen efficiency,^5^ and are readily oxidized by most organs, except the liver, which lacks acetoacetyl‐CoA thiolase, the enzyme required for the oxidation of ketone bodies via the tricarboxylic acid (TCA) cycle.^1^

Hyperpolarization using dynamic nuclear polarization (DNP) has revolutionized our ability to study non‐invasively metabolic changes in the living heart.^6^, ^7^ To date, the vast majority of hyperpolarization studies have focused on observing alterations in the metabolism of [1‐^13^C]pyruvate,^7^, ^8^ which provides unique insights into carbohydrate metabolism. However, alterations in the metabolic pathways of other key fuel molecules are a common feature of cardiovascular diseases,^9^, ^10^, ^11^ and it is known that ketone bodies exhibit a profound regulatory mechanism in controlling the use and release of other substrates, such as fat and glucose.^1^ The metabolism of acetoacetate and 3‐β‐hydroxybutyrate is known to be altered in various metabolic disease states, including diabetes and diabetic cardiomyopathy,^12^ and recalcitrant pediatric epilepsy has been clinically treated with a ketogenic diet for more than 70 years.^13^ The ability to study ketone body metabolism and its regulatory role *in vivo* may therefore offer new insights into the metabolic derangements seen in a range of disease states, and thus provide a new perspective on potential treatments.

In this work, we have investigated the use of hyperpolarized ^13^C‐labelled acetoacetate and β‐hydroxybutyrate as novel probes in the study of cardiac metabolism. Although several studies have shown the appearance of ketone bodies following the injection of related probes, such as [1‐^13^C]butyrate^14^, ^15^ and [2‐^13^C]pyruvic acid,^16^ the *in vivo* rate of appearance of ketone bodies is necessarily limited owing to organ specificity and the number of separate biochemical reactions required to produce them. The interpretation of the visible appearance (or lack thereof) of ketone bodies produced following the injection of these other probes is not necessarily straightforward and, in particular, the absence of evidence of a particular ketone body is not sufficient evidence of its absence. Such probes may therefore be considered to be inefficient for the study of ketone body metabolism, motivating the desire to look at the utilization of ketone bodies directly. We have therefore built on previous work^17^ and reported conference abstracts exploring the hyperpolarization of ketone bodies,^18^, ^19^, ^20^, ^21^ and report a method to generate hyperpolarized [1‐^13^C]acetoacetate and [1‐^13^C]β‐hydroxybutyrate. We further investigate the metabolism of [1‐^13^C]β‐hydroxybutyrate in the isolated, perfused rat heart, and [1‐^13^C]acetoacetate in both the perfused heart and the rat heart *in vivo*.

## 2 METHODS

*Ex vivo* experiments were performed using a Bruker Avance 11.7‐T vertical bore magnetic resonance imaging (MRI) system (Bruker Biospin GmbH, Ettlingen, Germany). *In vivo* experiments were performed using an Agilent 7‐T MRI system (Agilent, Santa Clara, CA, USA). DNP was performed using a HyperSense hyperpolarizer (Oxford Instruments, Abingdon, Oxfordshire, UK) for *ex vivo* experiments, or a previously described prototype hyperpolarizer^6^ (GE Healthcare, Amersham, Buckinghamshire, UK) for *in vivo* experiments. All animal investigations conformed to Home Office Guidance on the Operation of the Animals (Scientific Procedures) Act (HMSO) of 1986, to institutional guidelines and were approved by the University of Oxford Animal Ethics Review Committee. All compounds were obtained from Sigma Aldrich (Gillingham, Dorset, UK) unless otherwise specified.

### 2.1 Sample preparation

It was not possible to directly obtain free acetoacetic acid (or its sodium salt) owing to its chemical instability and comparatively short half‐life (approximately 2 h as an acid, several days as a base) at room temperature prior to spontaneous decarboxylation into acetone and carbon dioxide. Therefore, sodium [1‐^13^C]acetoacetate was produced by base‐catalysed hydrolysis of >99% isotopically labelled ethyl‐[1‐^13^C]acetoacetate.^22^ In this process, 250 μL of 8 M neat [1‐^13^C]ethyl acetoacetate was mixed with 4 mL of 1 M sodium hydroxide at 40°C for 6 h. Water was removed by lyophilization under reduced pressure (1 mbar) at –50°C for approximately 12 h, and the product (a white powder) was stored at –80°C.

Immediately prior to hyperpolarization, a stock solution was formulated by mixing 280 mg of the generated sodium [1‐^13^C]acetoacetate with 9.4 mg of OX063 trityl radical (Oxford Instruments), 50 μL of dimethyl sulphoxide (DMSO) and 150 μL of H_2_O. Similarly, a stock solution of [1‐^13^C]β‐hydroxybutyrate was formulated by mixing 280 mg of [1‐^13^C]β‐hydroxybutyrate (Sigma Aldrich; >98% purity and >99% ^13^C) with 9.4 mg of OX063, 50 μL of DMSO and 150 μL of H_2_O. Polarized samples were made by mixing 33 μL of either stock solution with 3.5 μL of 10mM Gd‐DOTA (Gd, gadolinium; DOTA, 1,4,7,10‐tetraazacyclododecane‐1,4,7,10‐tetraacetic acid; Dotarem, Guerbet, France) prior to vortexing and pipetting into a sample cup for rapid freezing. DNP was then performed at ~94 GHz and 100 mW for 60 min.^6^ Dissolution was carried out using 6 mL of heated and pressurized deionized water (~180°C, 10 bar), resulting in a final liquid state concentration of 40mM for both substrates. As a result of reagent and instrument availability, separate batches were synthesized for *in vivo*, *ex vivo* and phantom experiments. Deionized water was used to ensure that the compounds injected were kept under basic conditions, minimizing the spontaneous rate of decarboxylation occurring during the initial high‐temperature part of the dissolution process, which may otherwise lead to erroneously high recordings of bicarbonate originating from chemically evolved CO_2_ dissolved in buffer. The spontaneous rate of decarboxylation of acetoacetate is expected to obey an Arrhenius relation whose spectrophotometrically measured^23^ apparent first‐order rate constant is given by $k=\frac{k_{a}\left[H^{+}\right]+k_{i}K_{a}}{\left[H^{+}\right]+K_{a}}$, where *k*_a_ ≈ 29 × 10^−6^ s^−1^, *k*_i_ ≈ 0.5 × 10^−6^s^−1^ and *K*_a_ ≈ 2.84 × 10^−4^ which, at 37°C and pH 7, is ~5.5 × 10^–7^ s^–1^. Given that the rate of decarboxylation is expected to approximately double for each 10°C rise,^24^ we therefore only expect the spontaneous decarboxylation of acetoacetate to arise from exposure to temperatures in excess of ~150°C.

### 2.2 Polarization and *T*_1_ measurement

The liquid state polarization and *T*_1_ relaxation times were measured at 11.7 T. Two millilitres of hyperpolarized liquid were injected into a 20‐mm nuclear magnetic resonance (NMR) tube within a dual‐tuned ^13^C/^1^H probe (M2M Imaging, Cleveland, OH, USA) (*n* = 3). A ^13^C pulse and acquire sequence (4096 points; sweep width, 180 ppm; TR = 1 s) with 20 acquisitions at each of three different hard radiofrequency (RF) pulse durations (25, 50 and 75 μs), but constant RF power, was used to obtain spectra from the hyperpolarized sample. RF pulses were cycled in the order [10a, 10b, 10c, 10a, 10b, 10c], where a, b and c represent pulse durations of 25, 50 and 75 μs and ‘10a’ denotes 10 a‐degree pulses with a 1‐s TR. The measured signal decay was used to fit both *T*_1_ and flip angle to correct for the effects of RF excitation. The liquid state nuclear polarization was measured by comparison of the amplitude of the first point of the *T*_1_ decay curve with a subsequently acquired thermal polarization acquisition (TR = 300 s; averages, 24; flip angle, 90°). Signal amplitudes for the acquired spectra were fitted in the time domain using the AMARES algorithm in the jMRUI software package,^25^, ^26^ as described and illustrated further in the Supporting Information (Table S2; Figures S5–S8).

### 2.3 Isolated heart perfusion

Hearts from male Wistar rats (body weight, ~300 g; Harlan, Bicester, Oxfordshire, UK) were prepared for perfusion in the Langendorff mode, as described previously.^14^ Briefly, rats were terminally anaesthetized with 140 mg/kg pentobarbitone. Hearts were then excised and rapidly washed in ice‐cold Krebs–Henseleit (KH) buffer, followed by dissection to reveal the aorta. The aorta was cannulated, tied off with monofilament 3/0 (0.3 mm in diameter) silk suture (Pearsalls, Taunton, Somerset, UK) and the heart was perfused in the Langendorff mode at constant pressure (85 mmHg/11.3 kPa, corresponding to an approximate flow rate of 15 mL/min) using KH buffer at a temperature of 37°C and oxygenated with 95% O_2_/5% CO_2_ gas.^27^, ^28^, ^29^, ^30^ A polyethylene tube was inserted into the left ventricle and through the apex of the heart in order to drain Thebesian flow. A polypropylene balloon connected to a pressure transducer and a PowerLab system (AD Instruments, Abingdon, Oxfordshire, UK) was then inserted into the left ventricle to monitor contractile function, and the heart was subsequently placed inside a 20‐mm NMR test tube, which was inserted into the bore of the 11.7‐T magnet described above.

### 2.4 Assessment of ketone body metabolism in the perfused heart

Hearts from male Wistar rats (*n* = 3 per substrate, i.e. six rats in total) were initially perfused with KH buffer containing 10mM glucose. For the assessment of ketone body metabolism using hyperpolarized ^13^C substrates, dissolution of [1‐^13^C]acetoacetate and [1‐^13^C]β‐hydroxybutyrate was carried out, and the hyperpolarized liquid was added to a chamber containing oxygenated KH buffer. This generated a KH buffer with a final substrate concentration of 10mM glucose and 4mM hyperpolarized [1‐^13^C]acetoacetate or [1‐^13^C]β‐hydroxybutyrate. The supply to the perfused heart was subsequently switched to the hyperpolarized solution and a ^13^C pulse–acquire experiment was immediately started (TR = 1 s; hard pulse; sweep width, 180 ppm; 4096 complex points) with a 30° flip angle chosen as a compromise between the signal‐to‐noise ratio (SNR), intracellular metabolite signals and magnetization depletion, as discussed elsewhere.^31^, ^32^

### 2.5 Assessment of ketone body metabolism in the *in vivo* heart

Male Wistar rats (*n* = 4, distinct from the *ex vivo* study animals; body weight, ~300 g) were scanned in both the fed and subsequently overnight fasted metabolic states. To induce a fasted metabolic state, rats were kept in a 12‐h light/dark cycle and fasted for at least 12 h prior to scanning. A fed metabolic state was induced by scanning under the same conditions without overnight fasting, and with access to ‘standard chow’ *ad libitum*.

Anaesthesia was induced by 2.5–3% isoflurane in oxygen and nitrous oxide (2 L/min oxygen/isoflurane, 0.2 L/min nitrous oxide). Anaesthesia was maintained by means of 2% isoflurane delivered to, and scavenged from, a nose cone during the experiment. A tail vein catheter was placed for intravenous injection of hyperpolarized [1‐^13^C]acetoacetate. Animals were then placed in a home‐built animal handling system.^33^ Body temperature was maintained using air heating, and a two‐lead electrocardiogram (ECG) for cardiac gating was obtained using leads placed subcutaneously into the upper forelimbs. Blood ketone and glucose concentrations were measured via a commercially available hand‐held meter (Accu Chek, Bayer AG, Berlin, Germany) following injection of acetoacetate in a separate cohort of rats (*n* = 4).

^1^H images were acquired using a 72 mm‐inner‐diameter quadrature birdcage transmit/receive coil (Rapid Biomedical GmbH, Rimpar, Germany). ^13^C data were acquired using a 20 mm‐diameter ^13^C butterfly surface coil for signal transmission and reception. The surface coil was positioned on the anterior chest wall, and a thermally polarized 5 M ^13^C urea phantom was used to calibrate the ^13^C transmitter power before the ^13^C scans. A volume covering a 40‐mm slab including the heart was employed for shimming using a three‐dimensional gradient echo automated shim routine^34^ to reduce the proton linewidth to <1 ppm across the heart. Immediately prior to the injection of the hyperpolarized sample into the rat, an ECG‐gated ^13^C pulse–acquire sequence (nominal TR = 1 s; hard pulse of 15 μs; flip angle, 10°; sweep width, 6000 Hz; 2048 complex points; 60 measurements) was started, and 2 mL of hyperpolarized [1‐^13^C]sodium acetoacetate solution was injected over 10 s into the rat via the previously placed tail vein catheter. As documented previously, variation in the R–R interval of the anaesthetized rat yielded a slight variation in TR spacing (on the order of 1–10%) which was accurately measured using custom‐built hardware.^35^

### 2.6 ^13^C data analysis

All acquired ^13^C data were summed over a 60‐s period from the first spectrum in which hyperpolarized [1‐^13^C]acetoacetate or [1‐^13^C]β‐hydroxybutyrate could be clearly observed, and the summed spectra were fitted using the AMARES algorithm in the jMRUI software package. The AMARES algorithm was adequately constrained by prior knowledge of the spectral location of the numerous closely spaced resonances to enable their resolution. Fitted metabolite amplitudes were subsequently normalized to the maximum [1‐^13^C]acetoacetate/[1‐^13^C]β‐hydroxybutyrate amplitude to remove any contribution from polarization and injection timing differences between experiments. In addition, for kinetic analyses, a sliding window of length 5 (~5 s) was applied to the reconstructed data to improve the reconstructed SNR. Further information on spectral fitting is provided in Supporting Information.

### 2.7 Statistical methods

All data are given as mean ± standard error. Mean differences between fed and fasted states were assessed using an unpaired, unequal variance *t*‐test.

## 3 RESULTS

### 3.1 Liquid state polarization and longitudinal relaxation times

Hyperpolarization of [1‐^13^C]acetoacetate and [1‐^13^C]β‐hydroxybutyrate provided liquid state polarizations of 8 ± 2% and 3 ± 1%, respectively. The hyperpolarized *T*_1_ values for the two substrates were 28 ± 3 s (acetoacetate) and 20 ± 1 s (β‐hydroxybutyrate) at 11.7 T.

### 3.2 Metabolism of hyperpolarized ketone bodies within the isolated heart

**Figure 1:**
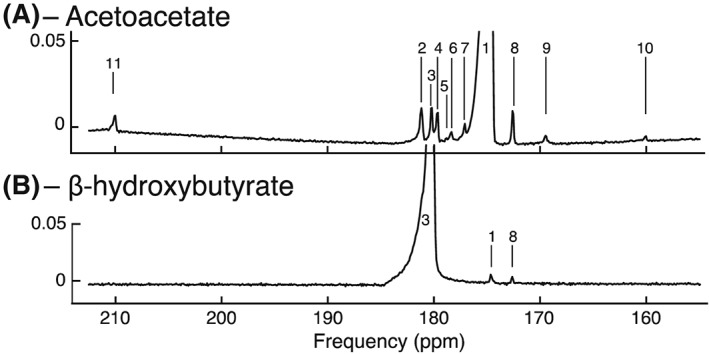
Ketone body metabolism in the perfused rat heart at 11.7 T. (A) Spectrum acquired following the administration of hyperpolarized acetoacetate. The observed resonances are: 1, [1‐^13^C]acetoacetate (175 ppm); 2, [5‐^13^C]glutamate + unknown (believed to be acetate impurity, 182 ppm); 3, [1‐^13^C]β‐hydroxybutyrate (181 ppm); 4, unknown (believed to be acetoacetate hydrate impurity, 180 ppm); 5, unknown (178.8 ppm); 6, [1‐^13^C]citrate (178.7 ppm); 7, unknown (177 ppm); 8, [1‐^13^C]acetyl‐carnitine (172.5 ppm); 9, [1‐^13^C]ethylacetoacetate (batch reagent impurity, 169 ppm); 10, ^13^C‐bicarbonate (161 ppm); 11, [3‐^13^C]acetoacetate (210 ppm). (B) Spectrum acquired following the administration of hyperpolarized [1‐^13^C]β‐hydroxybutyrate. The observed resonances are: 1, [1‐^13^C]acetoacetate; 3, [1‐^13^C]β‐hydroxybutyrate; 8, [1‐^13^C]acetyl‐carnitine. The *y*‐axis is shown relative to maximum peak intensity

Figure 1 shows the multiple downstream metabolites (and impurities) observed following the infusion of either hyperpolarized [1‐^13^C]acetoacetate (Figure 1A) or hyperpolarized [1‐^13^C]β‐hydroxybutyrate (Figure 1B) into the perfused rat heart. The resonances were identified using high‐resolution NMR [^1^H/^13^C and two‐dimensional heteronuclear multiple bond correlation (HMBC) performed at 7 T on a Bruker Avance 300 MHz spectrometer] subsequently acquired on chloroform/methanol metabolite extracts from snap‐frozen perfused heart tissue samples and with reference to the literature (shown as Supporting Information (Figures S1–S3; Table S1) with raw datasets provided as Supporting Information). Interconversion between the ketone bodies could be seen for both substrates, whereas infusion of hyperpolarized acetoacetate also allowed for the observation of metabolism into the Krebs cycle intermediates citrate and glutamate, together with the acetyl‐CoA buffering compound, acetylcarnitine. The infusion of ketone bodies did not significantly change the left ventricular developed pressure (LVDP), heart rate (HR) or the rate–pressure product (RPP) within the time course of the NMR experiment (shown in Supporting Information Figure S4). For 60 s prior to infusion, the mean ± standard deviation (SD) LVDP, HR and RPP were 80 ± 20 mmHg, 270 ± 30 bpm and 23 000 ± 8000 bpm mmHg, respectively; for 60 s following infusion, the corresponding means were 82 ± 16 mmHg, 270 ± 40 bpm and 22 000 ± 7000 bpm mmHg; *p* = not significant by a paired Welch (unequal variance) *t*‐test or via a non‐parametric Wilcoxon test.

The signals measured in the perfused rat heart indicated that downstream metabolism occurred to a greater extent following infusion of hyperpolarized acetoacetate, rather than β‐hydroxybutyrate. This was attributed to the lower *T*_1_ value and polarization level of [1‐^13^C]β‐hydroxybutyrate relative to [1‐^13^C]acetoacetate and, as such, β‐hydroxybutyrate was not further pursued as an *in vivo* probe. A small reagent impurity, ethyl‐[1‐^13^C]acetoacetate, was visible in the batch of synthesized [1‐^13^C]acetoacetate used for *ex vivo* experiments. Owing to the reported subsequent hepatic metabolism of ethyl‐[1‐^13^C]acetoacetate to ethanol and [1‐^13^C]acetoacetate,^36^ the presence of this impurity *ex vivo* was not considered to be significant for subsequent *in vivo* translation.

### 3.3 *In vivo* ketone body metabolism assessed using hyperpolarized [1‐^13^C]acetoacetate

**Figure 2:**
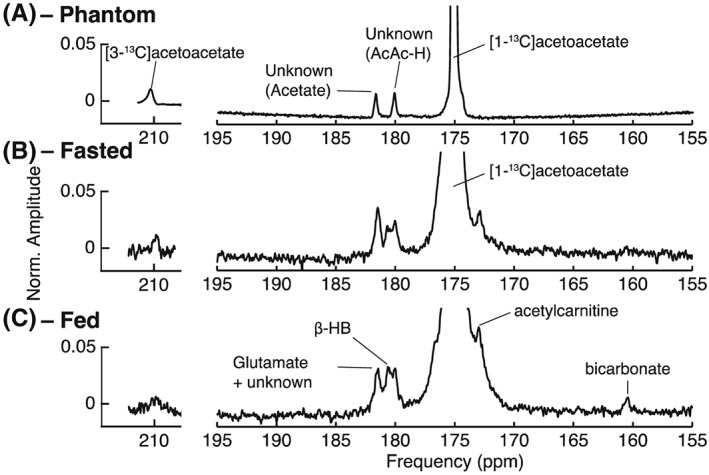
Summed spectra acquired following the administration of hyperpolarized [1‐^13^C]acetoacetate to the *in vivo* rat heart at 7 T. The displayed spectra are from (a) a phantom dissolution in water, (b) overnight fasted and (c) fed states. The resonances shown indicate conversion of the substrate [1‐^13^C]acetoacetate (175 ppm) into [1‐^13^C]acetylcarnitine (172.5 ppm) and [1‐^13^C]β‐hydroxybutyrate (181 ppm). Two resonances (180 and 182 ppm) are impurities in substrate production (top row), and that at 180 ppm is believed to be acetoacetate hydrate (AcAc‐H). The resonance at 182 ppm, which overlaps with [5‐^13^C]glutamate, has been denoted ‘glutamate’ in the text, and is believed to be acetate. Decarboxylation to [^13^C]‐bicarbonate (161 ppm) was observed only in the fed state (bottom row). The horizontal scale at ~210 ppm is expanded compared with that on the right; [3‐^13^C]acetoacetate is visible at 210 ppm. The ratio of [1‐^13^C]acetoacetate to [3‐^13^C]acetoacetate in phantom experiments is ~1%, as expected from natural abundance

**Figure 3:**
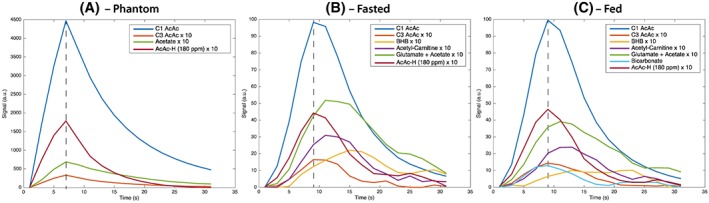
Representative kinetic time courses following the injection of hyperpolarized [1‐^13^C]acetoacetate after application of a sliding window averaging scheme, obtained following infusion of the agent into (a) a plastic phantom and in the (b) fasted and (c) fed states *in vivo*. Non‐acetoacetate peaks have been multiplied by a factor of ten. The dotted line present shows the point of maximum acetoacetate signal. The difference in the time‐to‐peak between injected acetoacetate and the glutamate + impurity peak is 0 s in the phantom experiment, rising to 2–4 s *in vivo*. We believe that this delay indicates that additional glutamate is produced underneath the impurity resonance. AcAc, acetoacetate; AcAc‐H, acetoacetate hydrate; BHB, β‐hydroxybutyrate

Figure 2 shows representative *in vivo* spectra acquired following the injection of 2 mL of 40mM [1‐^13^C]acetoacetate into fed and fasted rats. Impurities present in the substrate are also shown. The *in vivo* linewidth (0.5 ppm) was broader than in the isolated perfused heart as a result of cardiac and respiratory motion. Nevertheless, this linewidth was sufficient to resolve metabolism into acetylcarnitine as well as interconversion into β‐hydroxybutyrate. Citrate, located between [1‐^13^C]acetate and [1‐^13^C]acetoacetate at 178 ppm, was not observed, however, a small quantity of [3‐^13^C]acetoacetate at approximately 210 ppm was observed. This was believed to originate from the polarization of the natural abundance 3‐^13^C label in [1‐^13^C]acetoacetate. The main impurities in the substrate are believed to be [1‐^13^C]acetoacetate hydrate (~180 ppm) and [1‐^13^C]acetate (~182 ppm, which overlaps with the expected chemical shift of [5‐^13^C]glutamate). However, these impurity resonances were not uniquely identifiable following separate thermal equilibrium NMR experiments (cf. Supporting Information Figure S9), and are believed to originate spontaneously during the initial high‐temperature phase of the dissolution process used for *in vivo* experiments on prototype hardware. In separate phantom experiments at approximately 37°C, the apparent *T*_1_ value of the resonance at 182 ppm was seen to be the same as that of [1‐^13^C]acetoacetate (~30 s), indicating that it is in rapid exchange with acetoacetate (Figure 3). *In vivo*, this resonance decays with a different time course, with a prolongation consistent with metabolic production, and has a different relative amplitude to phantom experiments (in which the total integrated impurity to acetoacetate ratio had a maximum value of ~0.01), suggesting that the Krebs cycle product [5‐^13^C]glutamate is observable in addition to this impurity. If glutamate was not produced, but this resonance entirely reflected that of the impurity, we would expect it to have the same kinetic behaviour as the injected [1‐^13^C]acetoacetate. In phantom experiments, both peaks reach their maximum amplitude at the same time and in the same spectra. *In vivo*, however, the time to peak of ‘glutamate’ occurs 2–4 s after the maximum acetoacetate signal, which is consistent with metabolic production.

**Figure 4:**
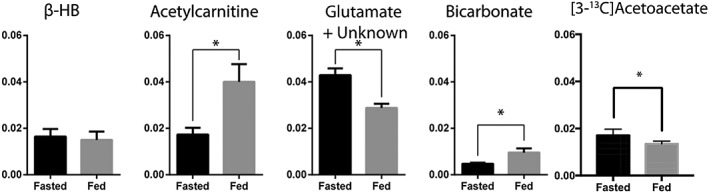
Metabolite ratios for β‐hydroxybutyrate (β‐HB), acetylcarnitine, glutamate and bicarbonate following the administration of hyperpolarized [1‐^13^C]acetoacetate to the *in vivo* rat heart. Statistically significant differences (*p* < 0.05) were observed in ^13^C‐labelled acetylcarnitine, glutamate + unknown, [3‐^13^C]acetoacetate and bicarbonate between fed and fasted states

Figure 4 shows the metabolite ratios following acetoacetate administration in the *in vivo* heart in the fed and fasted states. A statistically significant increase in the total [1‐^13^C]acetylcarnitine produced from the injected acetoacetate was observed in the fed state. In addition, a significant decrease in the level of the resonance at 182 ppm was observed in the fasted state. Decarboxylation of acetoacetate into bicarbonate was increased in the fed state compared with the fasted state (*p* < 0.05). The mean ratio of β‐hydroxybutyrate to acetoacetate was 0.0158 ± 0.005 in the fed state and 0.0163 ± 0.003 in the fasted state (*p* = not significant). A significant increase was observed in the [3‐^13^C]acetoacetate to [1‐^13^C]acetoacetate ratio between the fed (0.0135 ± 0.001) and fasted (0.017 ± 0.003) states (*p* = 0.04). No other significant differences were observed between the fed and fasted states. After infusion, the mean plasma ketone body concentration was 0.5 ± 0.1mM (fed) and 1.4 ± 0.2mM (fasted). Plasma glucose concentrations were 10.2 ± 2.3mM (fed) and 6.6 ± 0.35mM (fasted), neither of which were significantly different from the reported range under similar fasting conditions.^37^, ^38^, ^39^

## 4 DISCUSSION

**Figure 5:**
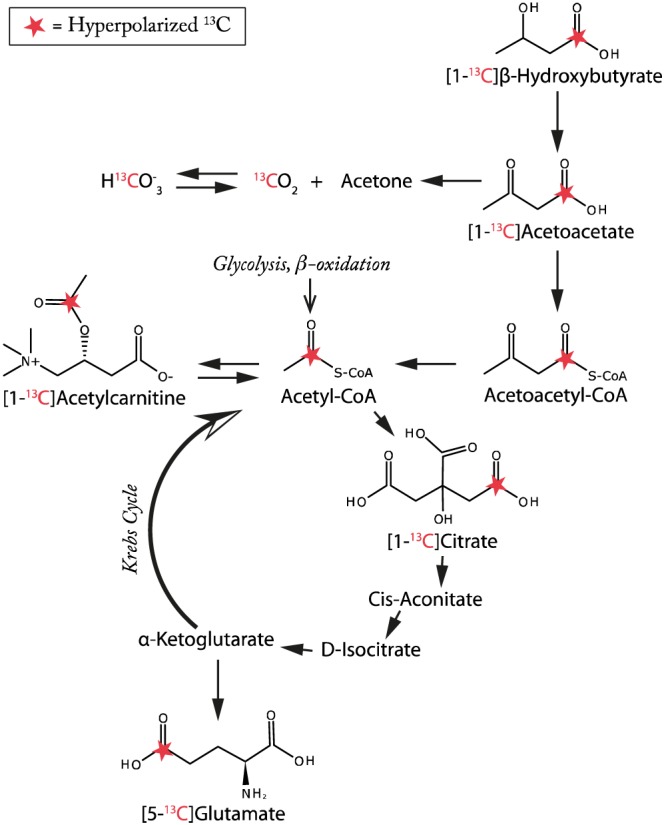
Summary of the metabolic pathways together with label positions believed to be probed by both hyperpolarized [1‐^13^C]acetoacetate and [1‐^13^C]β‐hydroxybutyrate in both the *in vivo* and *ex vivo* rat heart as presented in this work. Compared with the fasted state, acetylcarnitine production was increased, glutamate decreased and bicarbonate increased by feeding

Ketone body metabolism is altered in diseases affecting the heart, including diabetes and diabetic cardiomyopathy. For example, the sodium‐glucose co‐transporter‐2 inhibitor and novel anti‐diabetic drug, Empaglifozin, has been shown to dramatically reduce mortality and morbidity from cardiovascular causes in a large cohort study of patients with type II diabetes mellitus,^40^ and is believed to be associated with alterations in ketone body metabolism.^41^ A better understanding of the metabolic derangements seen in these diseases would provide a new perspective on potential treatments. This work has demonstrated the potential to hyperpolarize the ketone bodies acetoacetate and β‐hydroxybutyrate to a level sufficient to allow the observation of their metabolism in both the isolated perfused rat heart and, in the case of acetoacetate, the *in vivo* organ. The higher polarization level and longer *T*_1_ value achieved with acetoacetate enabled the observation of its rapid uptake, interconversion with β‐hydroxybutyrate and downstream metabolism into the Krebs cycle, as summarized in Figure 5. However, the spectral position of β‐hydroxybutyrate, in addition to the lower polarization level and shorter *T*_1_, meant that the reliable detection of the resonances of citrate and glutamate were not possible.

In the *in vivo* experiments, we observed significant differences in the metabolism of acetoacetate to acetylcarnitine, ‘glutamate’ and bicarbonate between the fed and fasted states (Figure 3). The apparent increased rate of acetylcarnitine production following feeding is consistent with the reported role of acetylcarnitine as a store of acetyl moieties should they be abundant in a post‐prandial state, into which ketone oxidation is directed.^42^ In the fasted state, ‘glutamate’ levels were higher, which is consistent with an increased flux of ketone bodies into the TCA cycle during fasting. However, further work is required on the synthesis methodology to prevent/remove the impurity that overlaps the glutamate resonance to confirm this finding, as we note that, owing to the presence of the impurity, the relative ratios of other metabolites to ‘glutamate’ are quantitatively dissimilar to those obtained through other methods.^15^ The ratio of β‐hydroxybutyrate to acetoacetate was apparently unchanged between the fasted and fed states, reflecting the unchanged appearance rate of exchange into β‐hydroxybutyrate; this may reflect on the constancy of the mitochondrial redox state during the relatively short overnight period of fasting undertaken in this experiment. The appearance of ^13^C‐bicarbonate following the injection of [1‐^13^C]acetoacetate was observed in both the perfused heart and *in vivo*. This is presumed to be caused by the spontaneous decarboxylation of acetoacetate to acetone and carbon dioxide. An alternative pathway is the *in vivo* decarboxylation of acetoacetate catalysed by acetoacetate decarboxylase, which is present within blood.^43^, ^44^ The time‐to‐peak intensity for this resonance was the same as that of acetoacetate itself, which indicates that production is rapid. Given that a delay of several seconds was observed between peak acetoacetate and peak acetylcarnitine and β‐hydroxybutyrate, this observation is likely to be consistent with a mechanism not involving transport across the plasma membrane.

Similar to Wang et al.,^17^ we observed [3‐^13^C]acetoacetate following the injection of acetoacetate synthesized from labelled precursors and expected to be labelled in the [1‐^13^C] position. We believe that there are two potential explanations for this observation: (1) the polarization of the natural abundance [3‐^13^C] label in [1‐^13^C]acetoacetate; and (2) a metabolic explanation that would necessitate multiple enzymatic steps and transport across the plasma and mitochondrial membrane. As the observed [3‐^13^C]acetoacetate resonance appears to have the same temporal behaviour (namely the same time‐to‐peak) as [1‐^13^C]acetoacetate, we consider that explanation (1) is more likely. In phantom experiments, the proportion of [3‐^13^C]acetoacetate is equal to that expected from hyperpolarized natural abundance carbon, but this is not the case *in vivo*. We hypothesize that this discrepancy may arise on two further grounds: (1) there may exist a difference in the *T*_1_ values of [1‐^13^C]acetoacetate and [3‐^13^C]acetoacetate in blood compared with in the phantom; or (2) owing to hardware imperfections and uncertainty in the centre frequency inherent in *in vivo* hyperpolarized experiments, we may have unwittingly introduced a slight difference in excitation bandwidth between the phantom and *in vivo* experiments. Interestingly, we observed a difference in [3‐^13^C]acetoacetate between the fed and fasted states, which we are at present unable to explain, but which represents an area of investigation for future work. We also note that Wang et al.^17^ did not observe any resonances beyond that ascribed to [1‐^13^C]acetate and [3‐^13^C]acetoacetate, and we hypothesize that this may be a result of the greater spectral separation achieved at 7 T than at 3 T. In the perfused heart at 11.7 T, multiple resonances were observed that would otherwise not be resolvable at lower field strengths.

Interestingly, we observed substantial ^13^C‐bicarbonate production almost exclusively in the fed state, but the *in vivo* significance of this result is not fully understood, although we note that this result is consistent with a difference in label exchange rates based on equilibrium positions, together with the observation in rodents that breath acetone is significantly increased by fasting or ketogenic diets.^45^ The spontaneous rate of decarboxylation would be expected to be independent of metabolic conditions, and proportional only to the (fixed) quantity of injected labelled compound. We therefore propose that increased bicarbonate production may reflect enzymatic blood decarboxylation, which is expected to be exceptionally rapid.^46^ In future studies, as well as mechanistically elucidating these changes, we will investigate differences in ketone body metabolism between baseline and diseased states (e.g. in models of type II diabetes), as well as during high cardiac workload. It may also be possible to monitor non‐invasively pathological changes in the mitochondrial redox state as indexed by the ratio between β‐hydroxybutyrate and acetoacetate.

In this proof‐of‐principle study, we observed ^13^C label exchange representing the metabolism of hyperpolarized [1‐^13^C]acetoacetate and [1‐^13^C]β‐hydroxybutyrate, whose labelling positions result in reasonable liquid state polarization and *T*_1_ values. However, the *in vivo* linewidth (0.5 ppm) of these probes following injection makes it challenging to distinguish some of the downstream products of [1‐^13^C]acetoacetate and, as such, other labelling positions may offer experimental advantages. In particular, [3‐^13^C]acetoacetate has a chemical shift of 210 ppm, upfield of the carbonyl resonances, although we note that flux through to citrate may introduce label exchange into the [1‐^13^C]position. In addition, the chemical instability of acetoacetate mandates the use of in‐house synthesis methods, which we have not fully optimized, leading to the production of two prominent impurity resonances that we believe are acetate and acetoacetate hydrate. Owing to the chemical instability of acetoacetate and the necessarily increasing time delay between batch synthesis and each individual experiment, despite our best efforts, it is likely that the exact impurity profile differs between each experiment. Nevertheless, we have demonstrated the regulation of ketone body metabolism in the rat heart, and have shown that the *in vivo* regulation of ketone body metabolism can be rapidly measured via dissolution DNP using a simple, accessible synthesis route. It should be noted that the chemical instability of the probe may make future translation to human studies challenging, although, if the compound is stored below 0°C, the rate of spontaneous decarboxylation is believed to be low, in part owing to its mechanism (reproduced in Supporting Information). Future work will continue to optimize this technique to improve the purity and yield of the synthetic route, and increase polarization, and also further explore ketone body metabolism in the pathophysiological heart with hyperpolarized MR, which is of critical importance in numerous disease states.

## 5 CONCLUSIONS

We report on the use of ^13^C‐labelled acetoacetate and β‐hydroxybutyrate as novel hyperpolarized substrates in the study of cardiac metabolism. Methods for the generation of hyperpolarized [1‐^13^C]acetoacetate and [1‐^13^C]β‐hydroxybutyrate were described, and their metabolism was investigated in both isolated, perfused rat hearts and in the *in vivo* rat heart.

## Supporting information

Data S1. Supplementary Material for Hyperpolarized Ketone Body Metabolism in the Rat Heart

Supporting info item
