# Supplementary material for: Hyperpolarized ketone body metabolism in the rat heart
Source: NMR Biomed. 2018 Apr 10;31(6):e3912. doi: 10.1002/nbm.3912 (PMC6001529; doi:10.1002/nbm.3912)
Supplement: Supplementary file 1 — Data S1. Supplementary Material for Hyperpolarized Ketone Body Metabolism in the Rat Heart [file NBM-31-na-s001.docx]

**Supplementary Material for Hyperpolarized Ketone Body Metabolism in the Rat Heart**

Jack J. Miller^1,2^, Daniel R. Ball^1^, Damian J. Tyler^1†^, and Angus Z. Lau^3†^

**High-resolution spectra**

Following perfusion, hearts were freeze-clamped in liquid nitrogen, and stored at -80 ºC. Metabolite extraction used a methanol/chloroform process, in which 200 µl of 2:1 methanol/chloroform was added to half a heart (~500 mg) crushed in liquid nitrogen using a ceramic pestle and mortar, and sonicated for 15 minutes. Subsequently 200 µl of water and 200 µl of chloroform were added, briefly vortexed and centrifuged at 13 500 rpm for 20 minutes. The aqueous layer was removed and air dried for approximately 2 hours. The resulting (yellow) extract was resuspended in 400 µl D2O in 250 mM sodium phosphate buffer (pH 7.4), vortexed, transferred to a 5 mm ø borosilicate glass NMR tube, and inserted into a Bruker Avance 300 MHz system.

Proton spectra were acquired using a water suppression pulse sequence (sw = 4789 Hz, ns = 1024, number of points = 16384, ppg=zg60pr) followed by a 2D HMBC acquisition correlating nuclei across multiple bonds (ns=256, ppg=hmbcgpndqf, sw=2635, 16762 Hz). Carbon spectra were then acquired for the remainder of the night (sw=17985, ns=16384, ppg=zpg30).

Peak assignment was performed on the basis of all available information and with reference to the NMR information in the human metabolite database [www.smpdb.ca](http://www.smpdb.ca). and [www.hmdb.ca](http://www.hmdb.ca), and are summarised below in table S1.

| Name | 13C (ppm) | 1H (ppm) |
| --- | --- | --- |
| C5-glutamate | 181.08 | 1.97, 2.19 |
| ß-hydroxybutyrate | 180.11 | 2.29, 2.26, 2.32 |
| Citrate | 178.25 | 2.47, 2.23 |
| Acetoacetate | 174.60 | 1.99, 3.84 |
| Acetyl carnitine | 172.52 | 2.03, 2.94 |

Table S1: Predominant metabolite peaks identified in high resolution NMR spectra obtained from tissue extracts

Spectra are shown below, in figures S1-S3.

Figure S1: High resolution 13C NMR obtained from chloroform/methanol heart extracts.

Figure S2: High resolution 1H spectra obtained from chloroform/methanol heart extracts, with parameters as given in the text.

Figure S3: Two dimensional proton/carbon HMBC obtained from heart extracts.

**Perfusion**

Injection of hyperpolarized β-hydroxybutyrate did not significantly change RPP, LVDP or the heart rate during the timecourse of the NMR experiment. An example pressure trace and corresponding computed heart rate (bpm), developed pressure (mmHg) and rate-pressure product (RPP) in the temporal region immediately prior to and following the injection of the hyperpolarised probe is shown in figure S4. The mean of all such quantities obtained 60 s prior to infusion was not significantly different from that obtained 60 s after the infusion, i.e. cardiac function was not changed within the timescale of the experiment.

Figure S4:Pressure, heart rate, developed pressure and RPP during a representative perfusion experiment, showing timings of the start and end of the NMR acquisition, together with the start location of the infusion of hyperpolarized ketone bodies.

**Spectral Processing**

Spectral data acquired at 500 MHz were DC corrected, and the initial ~63 points of the FID were truncated to remove an unblanking artefact created by the Avance Spectrometer used. They were then Fourier transformed, summed over time, referenced, quantified by AMARES and displayed apodized by a Lorentzian function (30 Hz linewidth). Data acquired at 300 MHz were quantified as above, but without initial point truncation. Representative illustrative spectral fits following summation for 60 s after infusion into a fed healthy rat, showing model FID estimated and experimentally measured peak amplitudes together with residuals, are shown in figures S5-6, together with returned model parameter values in table S2.


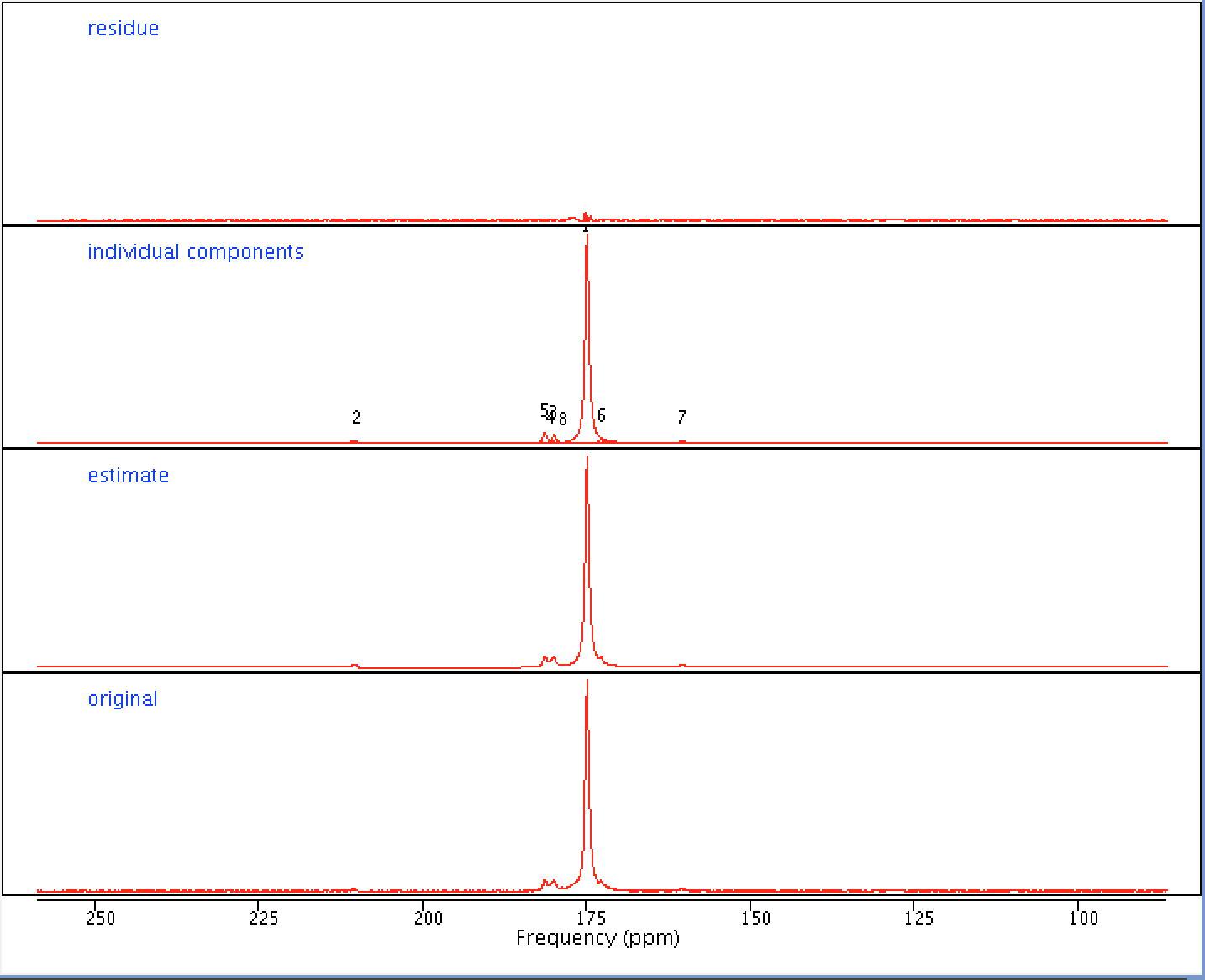


Figure S5: Illustrative examples of the AMARES fits to acquired data in jMRUI, shown at full scale.


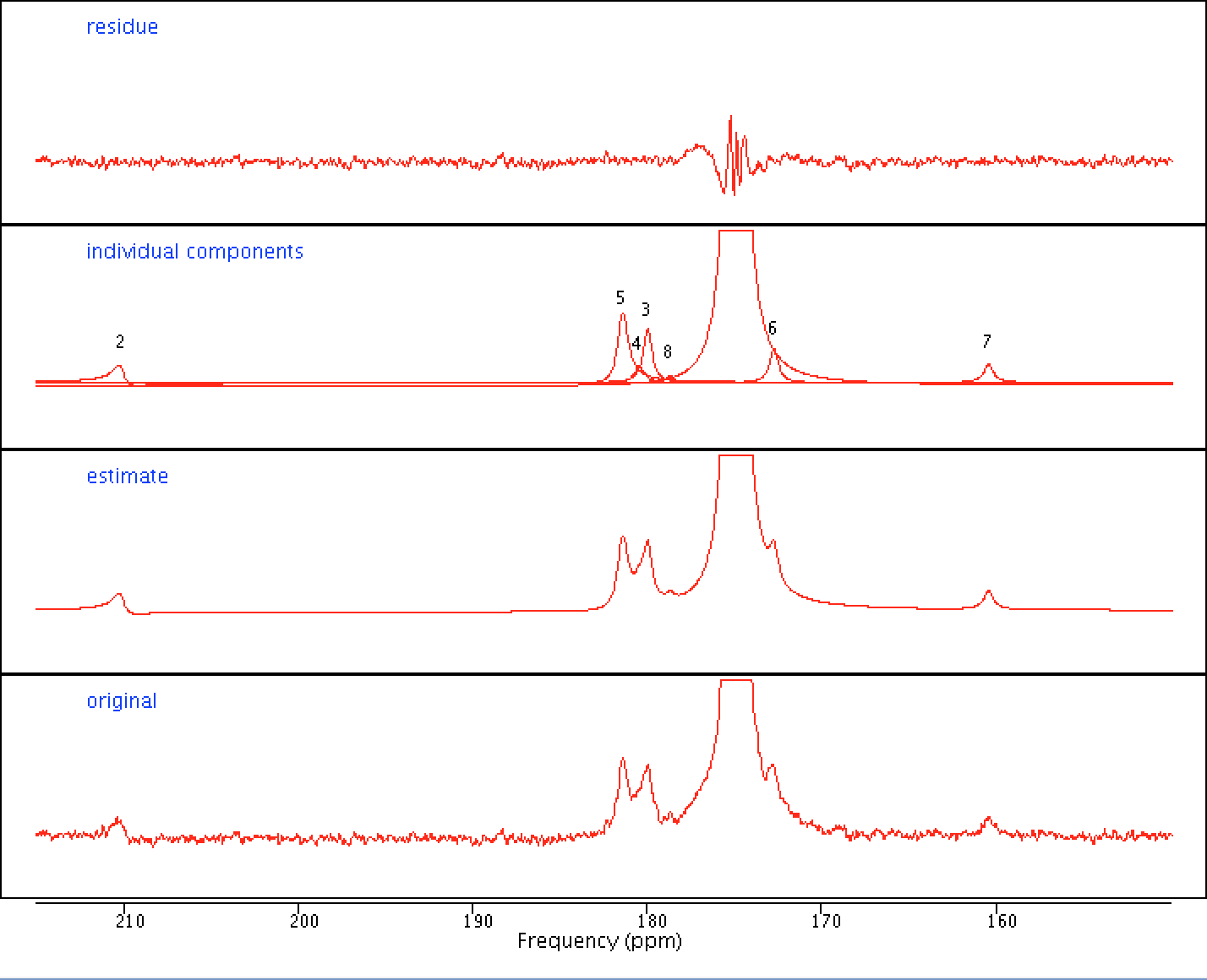


Figure S6: As per figure S5, but with the vertical scale zoomed in on the smaller metabolite peaks. With the exception of a slight deviation away from the Lorenzian lineshape of the injected probe, the remaining residuals are approximately normally distributed about 0.

| Name | Frequency (ppm) | Amplitude (au) | CRLB on Amp |
| --- | --- | --- | --- |
| 1: C1 AcAc  (injected probe) | 175.03 | 2.846e3 | 5.36 |
| 2: C3-AcAc (Polarised satellite) | 210.49 | 21.56 | 3.78 |
| 3: Acetate | 180.07 | 114.87 | 5.72 |
| 4: BHB | 180.62 | 33.73 | 5.80 |
| 5: Glutamate + Unknown | 181.48 | 136.16 | 4.17 |
| 6: Acetylcarnitine | 172.828 | 52.08 | 4.06 |
| 7: Bicarbonate | 160.54 | 25.10 | 3.78 |

Table S2:Returned AMARES parameters for the fit shown above.

It was found in all cases across all scans that peaks reported as being present had amplitudes very much greater than their corresponding Crámer-Rao Lower Bounds (CRLBs). We believe that the spectral fitting process is robust and the peaks found are appropriate. We note that this approach could be quantified by iterating over peaks included in the fitting algorithm and thence computing the BIC or AIC for model selection and optimisation, and a preliminary investigation into this question using a MATLAB based version of Amares that enables the computation of such quantities reveals that the number of peaks chosen for spectral fitting is optimal.

**Beta-Hydroxybutyrate phantom dissolution**

Similar to the acetoacetate experiments, a series of dissolutions were performed with hyperpolarized beta-hydroxybutyrate to permit the determination of T_1_ and estimate initial liquid state polarisation. In contrast to acetoacetate, no impurities were visible in phantom experiments. An example series of spectra and summed spectrum are shown for beta-hydroxybutyrate in figures S7-8.


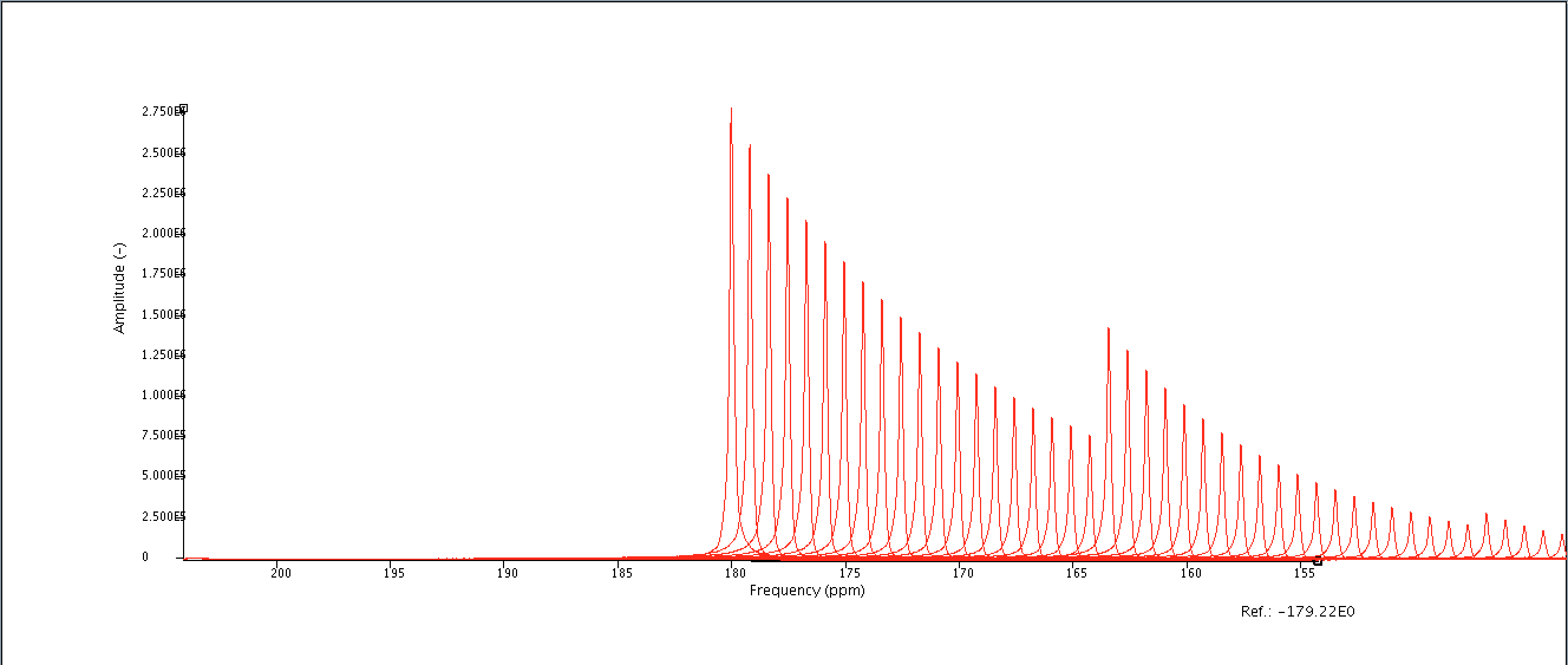


Figure S7:Timecourse of spectra acquired with three different flip angles showing the decay of hyperpolarised substrate; 1s TR.


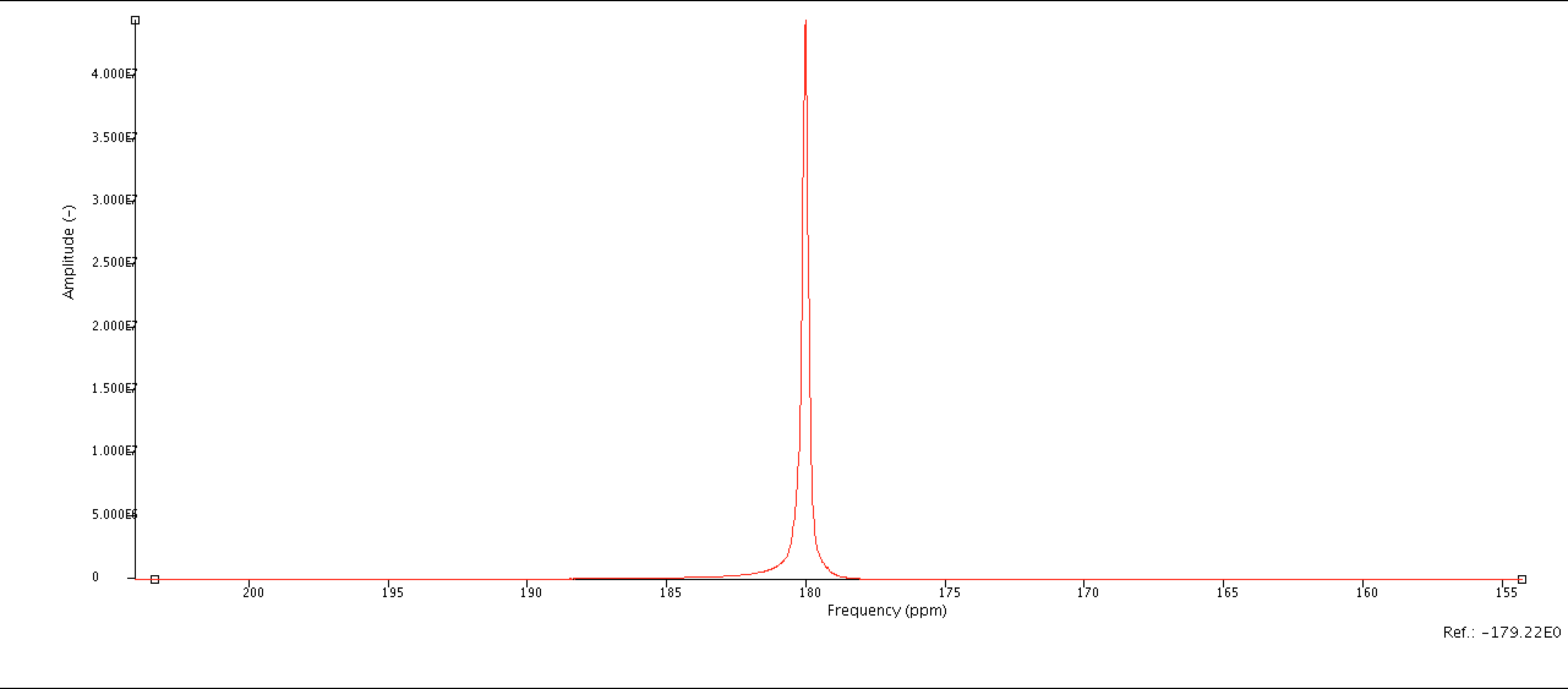


Figure S8:Summed spectra obtained from the phantom dissolution data shown in figure S7.

**Decarboxylation and impurity identification**

The spontaneous decarboxylation reaction is believed to proceed through a mechanism common to many keto-acids, namely:

where the red star indicates the 13C label. Under basic conditions, the carboxyl group is predominantly deprotonated and therefore is not expected to be able to form the stabilising coordinated six-membered ring shown. This is response for the increased half-life of acetoacetate under basic conditions, whereas under acidic conditions this step is much more likely to occur.

It is difficult to unambiguously identify the chemical identity of the two impurity resonances present in the acetoacetate spectra. Separate thermal-equilibrium high resolution proton and carbon NMR experiments on the dissolution product in a nearby institution unfortunately revealed that the majority of acetoacetate may have spontaneously decarboxylated during the time required to transport the sample to a nearby building in which the spectrometer was located; useful (i.e. non-acetone) carbon NMR spectra were additionally not obtainable. However, proton pulse/acquire spectra revealed peaks consistent with acetoacetate production as well as acetone and several broad resonances that are not easily identifiable (figure S9).

Figure S9: High resolution 1H NMR acquired on dissolution product.

We believe that these may be a polymeric structure and potentially acetoacetate hydrate, which is predicted to have a 13C chemical shift consistent with the observed labelled resonance at ~180 ppm. A plausible candidate dimer with a predicted chemical shifts consistent with experiment would be:

We note that comparable dimeric structures are well known to exist for similar small molecules, e.g. pyruvate and its acyclic dimers and oligomers.^[[1]](#footnote-1)^

1. C.f. S. A. Margolis and B. Coxon, “Identification and quantitation of the impurities in sodium pyruvate,” *Anal. Chem.*, vol. 58, no. 12, pp. 2504–2510, Oct. 1986. [↑](#footnote-ref-1)
