# Supplementary material for: Hyperpolarized ketone body metabolism in the rat heart
Source: NMR Biomed. 2018 Apr 10;31(6):e3912. doi: 10.1002/nbm.3912 (PMC6001529; doi:10.1002/nbm.3912)
Supplement: Supplementary file 2 — Supporting info item [file NBM-31-na-s002.zip › 1 (1H water sat)/pdata/1/email_Dec09-2011_1_1.pdf]

Instrument DPX300
Chemist Name DB
Research Group KC
13C 1,3 BHB trace (R)

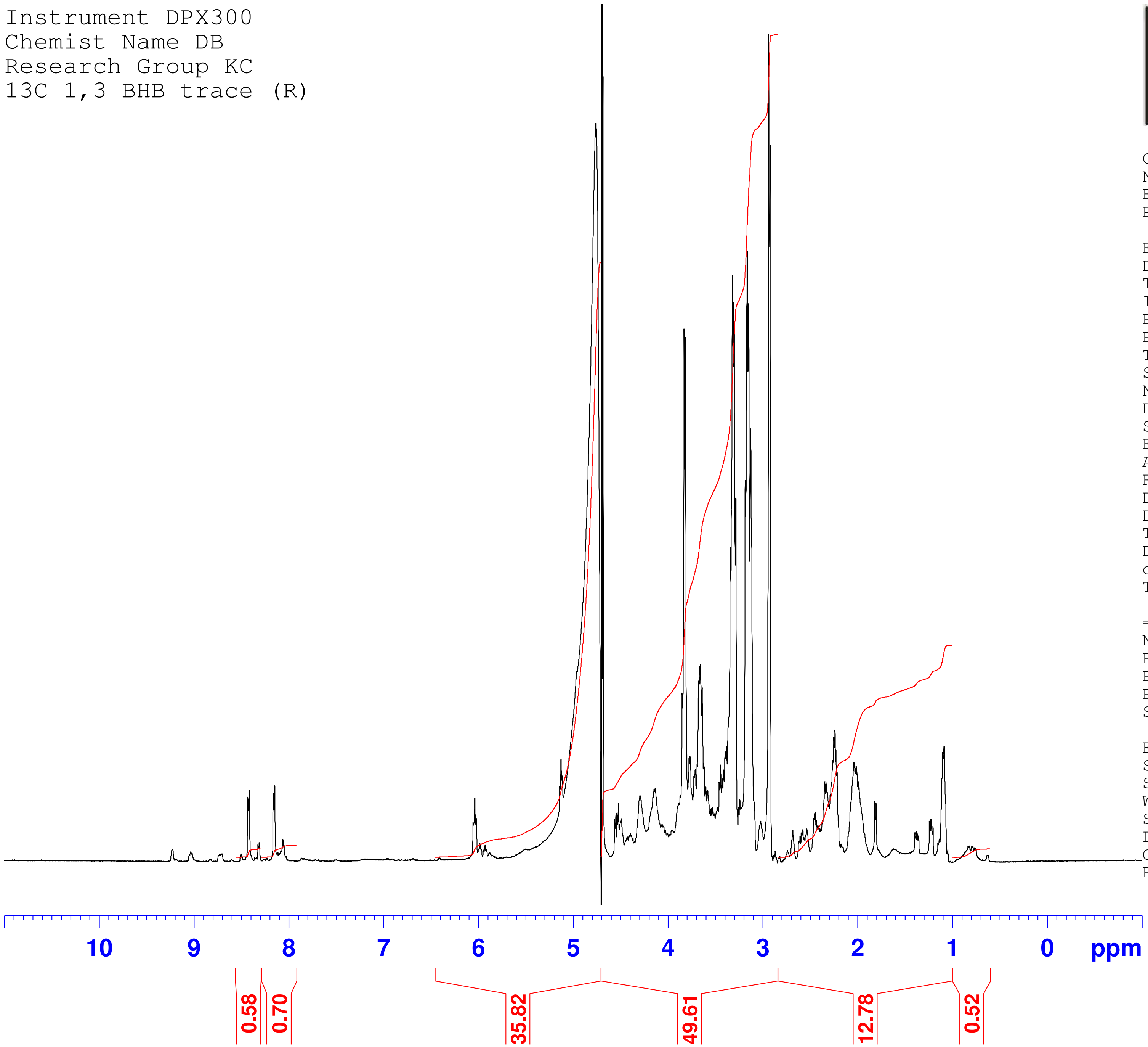

NMR@CHEM.OX

Current Data Parameters

NAME	Dec09-2011
EXPNO	1
PROCNO	1

F2 - Acquisition Parameters

Date_	20111209
Time	21.31
INSTRUM	DPX300
PROBHD	5 mm DUL 13C-1
PULPROG	zg60pr
TD	32768
SOLVENT	D2O
NS	1024
DS	2
SWH	4789.272 Hz
FIDRES	0.146157 Hz
AQ	3.4210291 sec
RG	512
DW	104.400 usec
DE	6.00 usec
TE	300.0 K
D1	2.00000000 sec
d12	0.00002000 sec
TD0	1

======== CHANNEL f1 ========

NUC1	1H
P1	16.50 usec
PL1	-6.00 dB
PL9	46.56 dB
SFO1	300.1314106 MHz

F2 - Processing parameters

SI	32768
SF	300.1300000 MHz
WDW	EM
SSB	0
LB	0.30 Hz
GB	0
PC	1.00
