# Supplementary material for: Hyperpolarized ketone body metabolism in the rat heart
Source: NMR Biomed. 2018 Apr 10;31(6):e3912. doi: 10.1002/nbm.3912 (PMC6001529; doi:10.1002/nbm.3912)
Supplement: Supplementary file 2 — Supporting info item [file NBM-31-na-s002.zip › 13C 1,3 BHB (16384 scans)/pdata/1/email_Dec12-2011_195_1.pdf]

Instrument DPX300
Chemist Name DB
Research Group KC
1,3 13C BHB tracing R

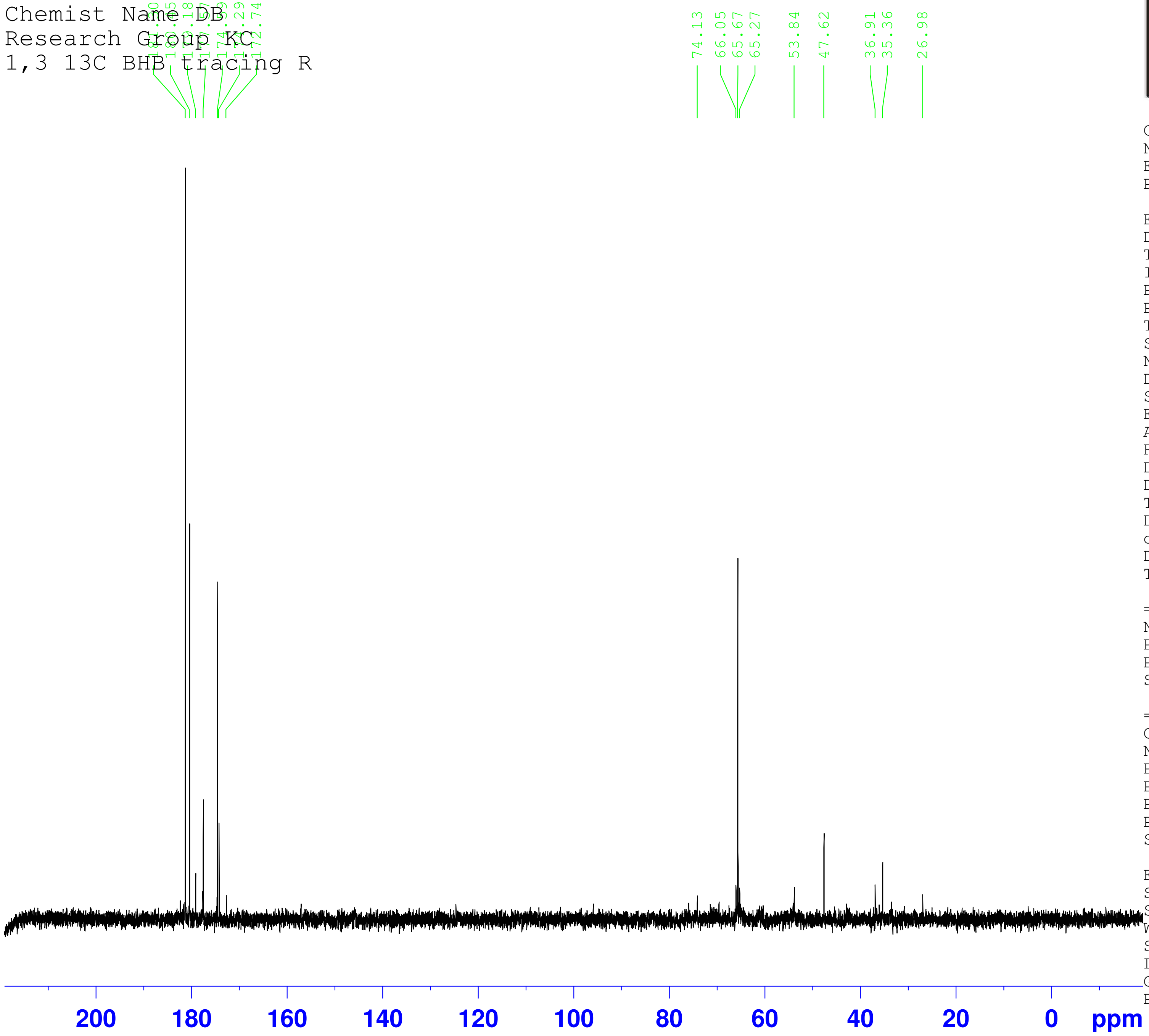

Current Data Parameters

NAME	Dec12-2011
EXPNO	195
PROCNO	1

F2 - Acquisition Parameters

Date_	20120108
Time	14.40
INSTRUM	DPX300
PROBHD	5 mm DUL 13C-1
PULPROG	zgpg30
TD	65536
SOLVENT	D2O
NS	16384
DS	4
SWH	17985.611 Hz
FIDRES	0.274439 Hz
AQ	1.8219508 sec
RG	14596.5
DW	27.800 usec
DE	6.00 usec
TE	300.0 K
D1	2.00000000 sec
d11	0.03000000 sec
DELTA	1.89999998 sec
TD0	1

======== CHANNEL f1 ========

NUC1	13C
P1	8.25 usec
PL1	-2.40 dB
SFO1	75.4752953 MHz

======== CHANNEL f2 ========

CPDPRG2	waltz16
NUC2	1H
PCPD2	80.00 usec
PL2	-6.00 dB
PL12	11.00 dB
PL13	11.00 dB
SFO2	300.1312005 MHz

F2 - Processing parameters

SI	32768
SF	75.4677490 MHz
WDW	EM
SSB	0
LB	1.00 Hz
GB	0
PC	1.40
