# Supplementary material for: Hyperpolarized ketone body metabolism in the rat heart
Source: NMR Biomed. 2018 Apr 10;31(6):e3912. doi: 10.1002/nbm.3912 (PMC6001529; doi:10.1002/nbm.3912)
Supplement: Supplementary file 2 — Supporting info item [file NBM-31-na-s002.zip › 2 (HMBC) 2/pdata/1/email_Dec09-2011_2_1.pdf]

Instrument DPX300
Chemist Name DB
Research Group KC
13C 1,3 BHB trace (R)

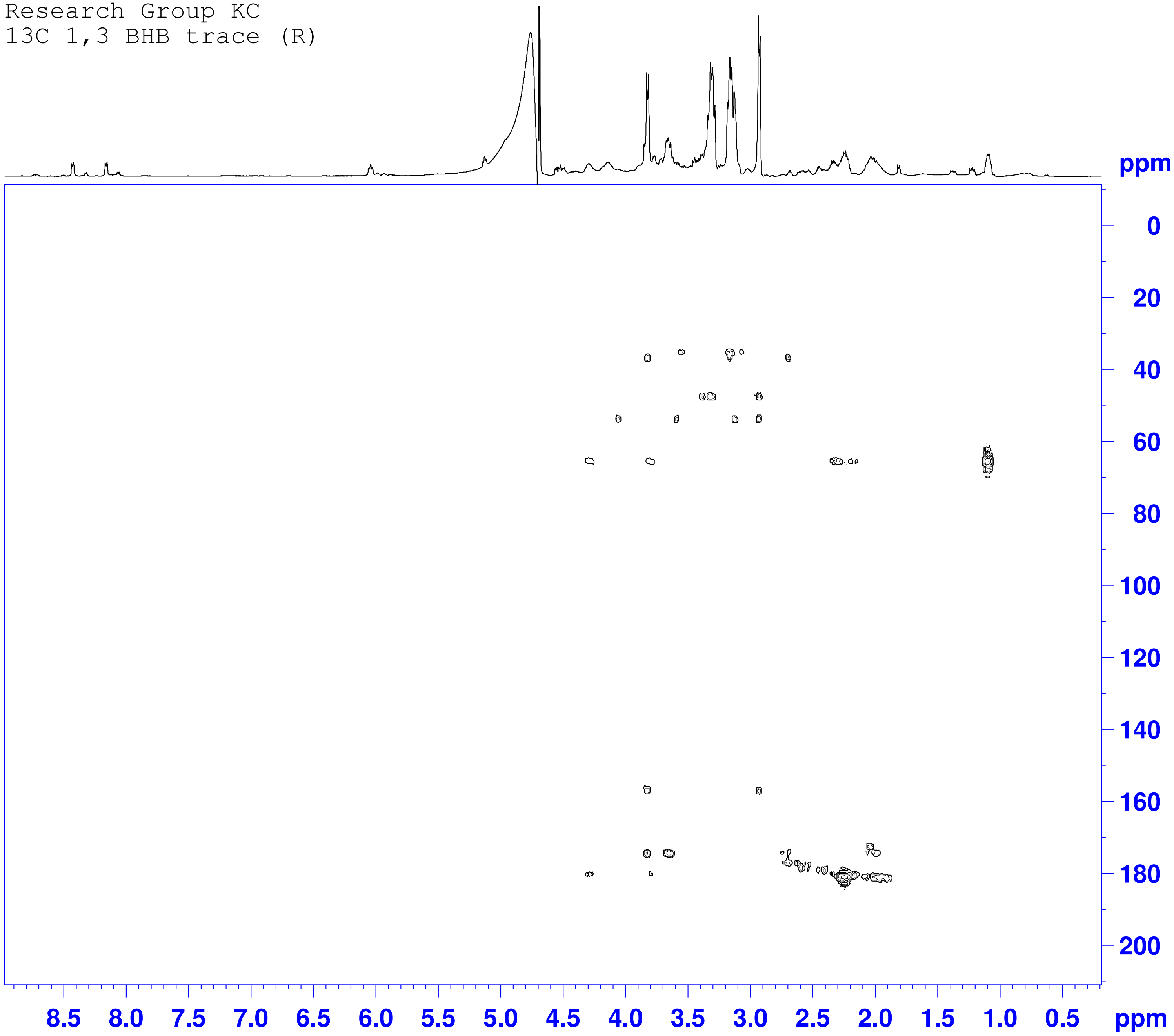


```
Current Data Parameters
NAME            Dec09-2011
EXPNO                    2
PROCNO                   1

F2 - Acquisition Parameters
Date_             20111209
Time                 21.40
INSTRUM             DPX300
PROBHD   5 mm DUL 13C-1
PULPROG        hmbcgpndqf
TD                    4096
SOLVENT                D2O
NS                     256
DS                      16
SWH               2637.131 Hz
FIDRES            0.643831 Hz
AQ               0.7766516 sec
RG                   26008
DW                 189.600 usec
DE                    6.00 usec
TE                   300.0 K
CNST13           8.0000000
d0              0.00000300 sec
D1              1.23294199 sec
d6              0.06250000 sec
D16             0.00010000 sec
IN0             0.00002980 sec

======== CHANNEL f1 ========
NUC1                    1H
P1                   16.50 usec
p2                   33.00 usec
PL1                  -6.00 dB
SFO1           300.1313748 MHz

======== CHANNEL f2 ========
NUC2                   13C
P3                    8.50 usec
PL2                  -3.50 dB
SFO2            75.4752833 MHz

====== GRADIENT CHANNEL =====
GPNAM1            SINE.100
GPNAM2            SINE.100
GPNAM3            SINE.100
GPZ1                 50.00 %
GPZ2                 30.00 %
GPZ3                 40.10 %
P16                1000.00 usec

F1 - Acquisition parameters
ND0                      2
TD                     128
SFO1              75.47528 MHz
FIDRES          131.082214 Hz
SW                 222.305 ppm
FnMODE                  QF

F2 - Processing parameters
SI                    1024
SF             300.1300000 MHz
WDW                   SINE
SSB                      0
LB                    0.00 Hz
GB                       0
PC                    1.40

F1 - Processing parameters
SI                    1024
MC2                     QF
SF              75.4677490 MHz
WDW                   SINE
SSB                      0
LB                    0.00 Hz
GB                       0
```
